# Near-Infrared Light Triggered the Shape Memory Behavior of Polydopamine-Nanoparticle-Filled Epoxy Acrylate

**DOI:** 10.3390/polym15163394

**Published:** 2023-08-13

**Authors:** Qi Wang, Xuefeng Yan, Ping Liu, Yiyan Xu, Qingbao Guan, Zhengwei You

**Affiliations:** 1 State Key Laboratory for Modification of Chemical Fibers and Polymer Materials, College of Materials Science and Engineering, Institute of Functional Materials, Research Base of Textile Materials for Flexible Electronics and Biomedical Applications (China Textile Engineering Society), Shanghai Engineering Research Center of Nano-Biomaterials and Regenerative Medicine, Donghua University, Shanghai 201620, China; wangqi@hexin-puleather.com (Q.W.); sudalp@yeah.net (P.L.); zyou@dhu.edu.cn (Z.Y.); 2 Zhejiang Hexin New Material Co., Ltd., Jiaxing 314000, China; yanxuefeng771082@163.com

**Keywords:** shape memory, near-infrared, photothermal, polydopamine, epoxy acrylate

## Abstract

Through the effective combination of photothermal conversion agent polydopamine (PDA) nanoparticles and epoxy acrylate polymer (EA), a new kind of near-infrared (NIR) light-triggered shape memory polymer (PDA/EA) is developed. Due to the outstanding photothermal effect of PDA, even with a very low concentration of PDA (0.1 wt.%), when exposed to an 808 nm NIR light with a power of 1 W/cm^2^, the temporary shapes can be fully light-responsive, recovered in 60 s. Based on dynamic thermomechanical analysis and thermal gravimetric analysis, it can be seen that the introduction of PDA is beneficial for improving dynamic mechanical properties and thermal resistance compared to EA. As an environmentally friendly and highly efficient photoactive SMP, PDA/EA has a great application prospect.

## 1. Introduction

Shape memory polymers (SMPs) are a class of intelligent materials that, in response to a specific stimulus, may revert to their original shape from a temporary shape [1,2,3]. The existence of thermal transitions, such as glass transition (*T_g_*), melting (*T_m_*), and liquid-crystalline transition (*T_LC_*) temperatures, allows the majority of SMPs to complete the shape memory process. When heated above these transition temperatures (*T_trans_*), the polymer is deformed and obtains a temporary shape; upon cooling, the shape will be fixed; when heated above *T_trans_* again, the polymer chains are allowed to relax and release internal stress; eventually, the polymer recovers back to its original shape [4].

Light is a far better stimulation source than heat. First, it could be remotely controlled, properly focused, and swapped quickly. Second, light’s wavelength and intensity may be altered to meet various needs [5,6]. In general, photo-responsive SMPs can be obtained through bringing in photo-isomerisable chemical groups or doping photothermal conversion agents based on photothermal effect [7,8,9]. Near-infrared (NIR) light has excellent tissue penetration ability, which is safer for human beings than UV/vis. Inorganic nanofillers possessing high NIR absorption capacity, such as gold nanorods or nanoparticles, graphene and carbon nanotubes, are widely used as photothermal conversion agents [10,11,12]. However, there is always a difficulty with the interfacial interaction between the polymer and the inorganic fillers, which may impair the original mechanical or thermal properties of the polymer. Thus, developing a new NIR-triggered SMP based on an efficient photothermal conversion agent/polymer system without sacrificing its integrated properties is very significant.

Mussel-inspired polydopamine (PDA) was firstly reported as a smart coating material by Messersmith et al. [13]. Research has revealed that dopamine-melanin colloidal nanospheres exhibit an efficiency 100 times greater than carbon nanotubes in converting NIR into heat [14]. Li et al. reported on the development of a PDA-coated SMP. This innovative coating enabled shape recovery that can be triggered by light, as well as the light-controlled shape reprogramming and surface functionalization of polymers [5]. The chemical structure of PDA, which contains several functional groups, is another characteristic. According to Yang et al., PDA was disseminated in commercial shape memory polyurethane (SMPU) and created a solid interface connection with the SMPU segments, which improved the mechanical characteristics [15]. Therefore, it is meaningful to choose PDA as a photothermal conversion agent to fabricate light-responsive SMPs.

In this study, we aim to propose a universally applicable approach for the fabrication of NIR-stimulated SMPs through dispersing PDA nanoparticles into ultraviolet (UV)-curable epoxy acrylate resin (EA). EA has high transparency and low viscosity, so it is easy to mix with nanomaterials, and can be quickly cured under ultraviolet (UV) light. A rapid rise in the temperature of PDA/EA can be induced upon NIR irradiation to realize shape recovery at a low concentration of PDA (0.1 wt.%) due to its outstanding photothermal effect. On the basis of dynamic thermomechanical and thermal gravimetric measurements, it can be seen that PDA/EA not only exhibits an excellent NIR-responsive shape memory effect, but also offers good thermal stability and mechanical properties.

## 2. Experimental Section

### 2.1. Materials

3-Hydroxytyramine hydrochloride (DOPA, 99%) was purchased from Alfa Aesar Chemical Co., Ltd., Shanghai, China. Iron (III) chloride hexahydrate (99%) was obtained from Shanghai Aladdin Biochemical Technology Co., Ltd., Shanghai, China. Tris(hydroxymethyl)aminomethane (Tris, 99.5%) was bought from Beijing Bellingway Technology Co., Ltd., Beijing, China. Epoxy resin was provided by Nantong Xingchen Synthetic Material Co., Ltd., Nantong, China. 4-Methoxyphenol (MEHQ, 98%) was purchased from Shanghai Yuanye Biological Technology Co., Ltd., Shanghai, China. Tetramethylammonium chloride (TMAC) was obtained from Sinopharm Group Chemical Reagent Co., Ltd., Shanghai, China. Acrylic acid was purchased from Shanghai Lingfeng Chemical Reagent Co., Ltd., Shanghai, China. Ethylene glycol diglycidyl ether was bought from Ailan (Shanghai) Chemical Technology Co., Ltd., Shanghai, China. 2,4,6-Trimethyl benzoyldiphenyl phosphine oxide (TPO, 98%) was provided by TCI (Shanghai) Chemical Industry Development Co., Ltd., Shanghai, China.

### 2.2. Preparation of PDA Nanoparticles

45 mg of DOPA and 1.3 mg of iron (III) chloride hexahydrate were completely dissolved in 130 mL of deionized water under stirring at room temperature for 1 h. A quantity of 20 mL of Tris aqueous solution (180 mg of Tris and 20 mL of deionized water) was quickly injected into the established solution. It could be observed that the solution color immediately turned red. Gradually, the solution color turned black after 0.5 h; this was followed by further stirring for 1.5 h. The targeted PDA nanoparticles were washed with deionized water three times and obtained from freeze drying after centrifugation.

### 2.3. Preparation of EA Oligomer

**Figure 1:**
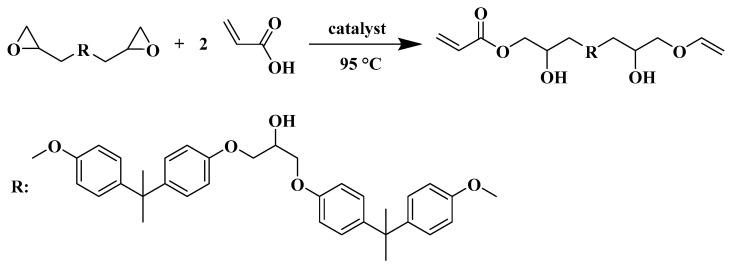
The synthesis route of EA oligomer.

Epoxy resin (1 mol) and MEHQ (0.1 wt.% of epoxy resin) were charged into a flask under a nitrogen atmosphere. The mixture was heated to 70 °C under stirring. TMAC (1 wt.% of epoxy resin) and acrylic acid (1.8 mol) were added drop wise in 0.5 h to obtain a colorless and clear liquid mixture. The mixture was heated to 95 °C and kept under stirring until the acid value of the mixture was less than 5 mg KOH/g. Then, the obtained yellow viscous product was EA oligomer. The synthetic route is shown in Figure 1. FTIR (KBr, cm^−1^): 3453 (–OH), 1725 (C=O), 1634 and 1408 (CH_2_=CH– of acrylate), 1510 (phenyl).

### 2.4. Synthesis of UV-Cured EA and EA/PDA Polymers

The formulations of EA/PDA polymers are listed in Table 1. An appropriate amount of EA oligomer, PDA, TPO (1 wt.% of EA oligomer), and ethylene glycol diglycidyl ether were evenly blended with mechanical stirring at 25 °C for 0.5 h to form a uniform polymer, which was then degassed under vacuum. The polymer was poured into the glass mold and cured in UV-curing equipment (QUV/spray, Q-LAB, Westlake, OH, USA) with a power intensity of 0.89 W/cm^2^. The UV irradiation process of polymer was carried out under an air atmosphere for 5 min.

### 2.5. Characterizations

Fourier-transform infrared (FTIR) spectra were recorded in a wavelength range of 400 to 4000 cm^−1^ with a resolution of 2 cm^−1^ on a Nicolet 6700 spectrometer (Thermo Fisher, Waltham, MA, USA).

A scanning electronic microscope (FE-SEM S-4700, Hitachi, Tokyo, Japan) was employed to study the morphology of PDA nanoparticles.

Thermogravimetric analysis (TGA) was carried out on a SDT2960 (TA instrument, New Castle, DE, USA) in a range of 50 to 600 °C with a heating rate of 10 °C/min under a nitrogen atmosphere.

Dynamic mechanical analysis (DMA) was performed on a DMA Q800 (TA instrument, New Castle, DE, USA) in tension mode. DMA tests were carried out from −40 to 120 °C using a frequency of 1 Hz, at a heating rate of 3 °C/min.

**Figure 2:**
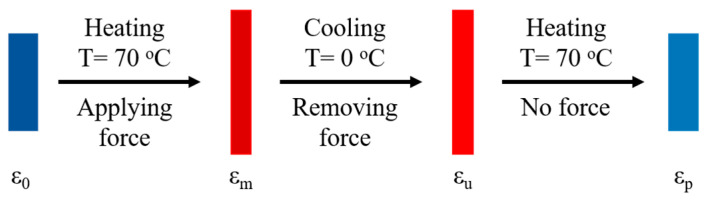
Schematic illustration of a shape deformation, fixation, and recovery cycle of the dual SM process in tension mode.

The shape memory (SM) thermomechanical cycle tests were studied via DMA Q800 (TA instrument, New Castle, DE, USA). The shape fixation (*R_f_*) and recovery efficiency (*R_r_*) were determined from SM cycles carried out in controlled tension mode. The dimensions of rectangular thin films were (15.0 ± 1.0) mm × (5.0 ± 0.5) mm × (0.5 ± 0.1) mm. The procedure for the dual SM tension test (Figure 2) included the following steps: (1) heating the sample to 70 °C from room temperature and isothermal holding for 5 min; (2) elongating the heated sample to a predetermined strain as its initial shape (*ε_m_*) at a constant elongation rate of 1%/min; (3) keeping the stress and cooling the sample to 0 °C at a rate of 5 °C/min to fix the polymer chains; (4) holding for 10 min at 0 °C to get a fixed shape (*ε_u_*) before the unloading of the stress; (5) reheating the sample to 70 °C at a rate of 5 °C/min, allowing the sample to relax to a recovery shape (*ε_p_*).

**Figure 3:**
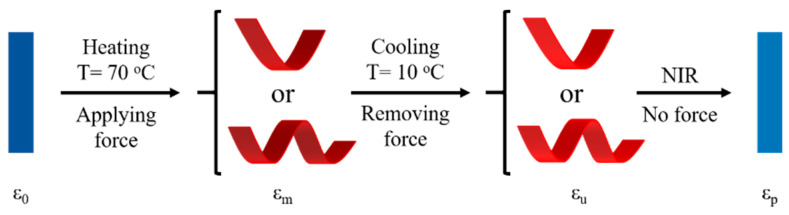
Schematic illustration of NIR-triggered SM process of EA/PDA polymers.

The real-time recording photothermal effect and NIR-triggered SM of EA/PDA resins were conducted using an infrared thermal imager (Fotric 225, Shanghai, China). The study also examined the photothermal effect and NIR-triggered SM via NIR light at a wavelength of 808 nm. The NIR-triggered SM process of EA/PDA polymers was illustrated in Figure 3. For instance, EA/0.1PDA was initially deformed and subsequently fixed through a heating and cooling process while being held under strain. Upon NIR irradiation at an intensity of 1 W/cm^2^, the deformed shape can recover to the original one.

## 3. Results and Discussion

### 3.1. Preparation of EA/PDA

**Figure 4:**
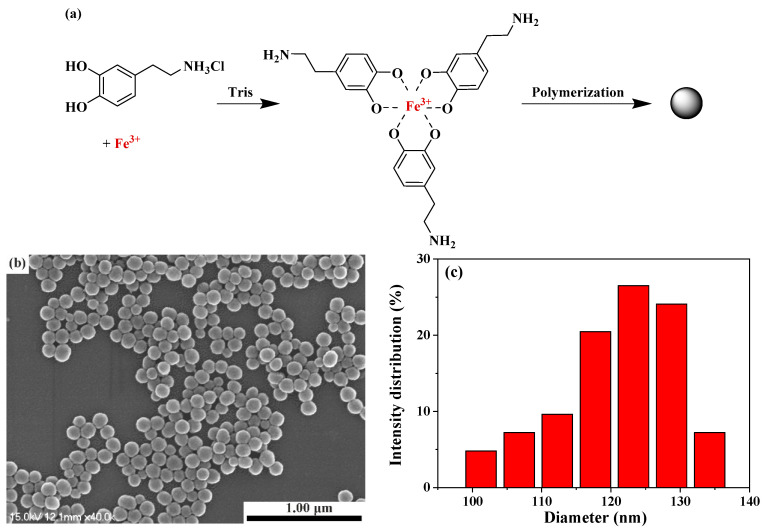
(**a**) Schematic illustration of the synthesis of PDA nanoparticles. (**b**) SEM image of PDA nanoparticles. (**c**) PDA nanoparticles diameter distribution.

PDA nanoparticles were synthesized through the oxidation polymerization of dopamine in a weak alkaline aqueous solution at room temperature, as illustrated in Figure 4a [16]. During the polymerization process, the dopamine was spontaneously oxidized and polymerized, resulting in the formation of spherical PDAs through intra/intermolecular cross-linking. The ferric ions within the nanospheres primarily formed coordination bonds with the catechol groups present in PDA. Notably, as the feed ratio of ferric ion to DOPA decreased, the final morphology of the Fe(III)-PDA complex transitioned from sheet-like to spherical [17]. SEM shows that the resultant PDA nanoparticles are spherical with uniform size distribution (Figure 4b). Nano Measurer 1.2 was employed to statistically analyze the particle size distributions based on the SEM images, revealing an average particle size of approximately 120 nm (Figure 4c). The SEM image of the fracture surfaces of UV-cured EA/PDA polymer indicated that the good dispersion of PDA nanoparticles in the EA matrix, as shown in Figure S1.

### 3.2. Thermal Properties of Cured EA/PDA

**Figure 5:**
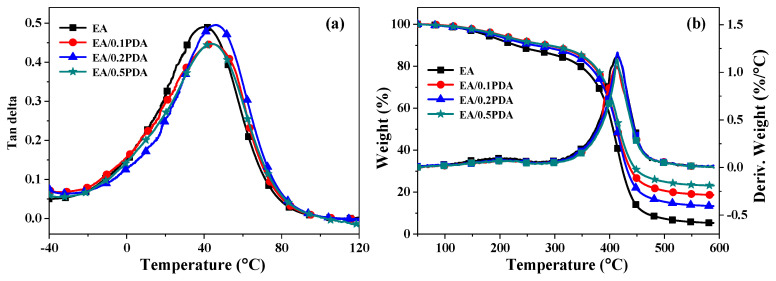
Thermal properties of UV-cured EA and EA/PDA polymers. (**a**) Plot of tan *δ* as a function of temperature for each polymer. Heating rate 3 °C/min and a frequency of 1 Hz. (**b**) TG and DTG curves of each polymer. Heating rate of 10 °C/min.

The glass transition temperature (*T_g_*) and thermalgravimetric (TG) behavior are two important parameters to evaluate the thermal properties of polymers, while *T_g_* represents the uppermost temperature at which a material can maintain its structural integrity, and TG behavior indicates the ability of polymers to withstand thermal stress over time. The *T_g_* value is defined by the peak temperature in the curve of tan *δ* as a function of the temperature obtained from DMA measurement (Figure 5a and Table 2). Cured EA shows a peak at 40 °C, whereas with the introduction of PDA nanoparticles into the EA matrix, the peak slightly shifts to a higher temperature (*T_g_* = 42–45 °C) for cured EA/PDA polymers. The result implied that the functional groups present in PDA (such as catechol, amine, and imine) undergo a reaction with the EA matrix, leading to the formation of additional cross-linking structures and resulting in a limitation of molecular chain segment activity.

The TG and DTG curves of cured EA/PDA and EA polymers are depicted in Figure 5b. Table 2 provides a summary of the initial degradation temperature (*T_di_*), which is defined as the temperature when the sample experiences a weight loss of 5 wt.%, as well as the temperature corresponding maximum degradation rate (*T_dmax_*) and char yield (*Y_c_*) at 600 °C. With the addition of PDA into EA matrix, the cured EA and EA/PDA polymers have similar shape of DTG curves and *T_dmax_* value (413 ± 2 °C), implying that the incorporation of PDA does not significantly affect the thermal degradation mechanism. It is noteworthy that the *T_di_* and *Y_c_* values of EA/PDA polymers are dramatically increased. TG and DTG results suggest the formation of strong interfacial interaction between PDA nanoparticles and the EA matrix instead of simply dispersion; thus, the thermal stability of resultant EA/PDA polymers can be effectively improved with a very low concentration of PDA (0.1–0.5 wt.%).

### 3.3. Thermomechanical Properties of Cured EA/PDA

**Figure 6:**
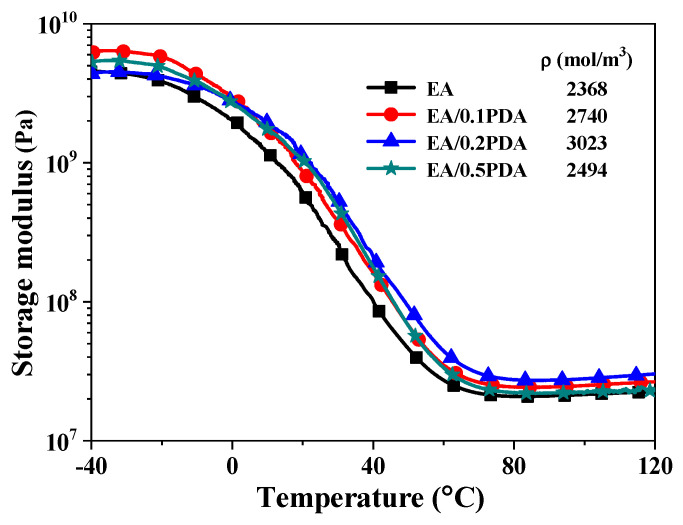
Plots of storage modulus as a function of temperature for UV-cured EA and EA/PDA polymers. Heating rate 3 °C/min and a frequency of 1 Hz.

The storage modulus is an important parameter for reflecting the stiffness of polymers. The cured EA/PDA polymers show a larger storage modulus than EA (Figure 6), indicating that the introduction of PDA nanoparticles is beneficial for improving the stiffness of EA, which results from the strong interfacial interaction between PDA nanoparticles and the EA matrix. More importantly, the plot of storage modulus as a function of temperature for each polymer shows a distinct step change from the glassy state to the rubbery state (Figure 6), which is beneficial for fixing the temporary shape with chemical crosslinked network and recovering to the original shape with high elasticity. Thus, the thermal-responsive shape memory (SM) behavior of resultant EA/PDA within a certain temperature range (~40 °C) above *T_g_* can be expected.

The overall performance of a thermosetting polymer is greatly influenced by its structural composition, including the polymer backbone and crosslinking network. Precisely, the extent of crosslinking can be quantified using the crosslinking density (*ρ*). Through utilizing the classical equation derived from the statistical theory of rubber elasticity (Equation (1)), it is feasible to determine the *ρ* values for cured EA and EA/PDA polymers.

(1) $$\rho =\frac{E^{\prime }}{3RT}$$

where E′ is the storage modulus of the cured polymers at rubbery plateau at a temperature (*T*) that is 40 °C higher than *T_g_*; *R* is the gas constant. The corresponding *ρ* values of cured EA and EA/PDA polymers are presented in Figure 6. Cured EA/0.5PDA polymers exhibit a larger *ρ* value (2494 mol/m^3^) than that (2368 mol/m^3^) of cured EA, further confirming that the incorporation of PDA slightly enhanced the crosslink reaction of EA due to the presence of functional groups (–OH and –NH_2_) of PDA nanoparticles. Overall, these EA/PDA composites are not highly crosslinked systems.

### 3.4. Thermal Responsive SM Behavior

Because of the distinct step change of storage modulus with temperature for each polymer, the glass transition process can serve as the thermal responsive switch for dual-shape memory behavior. Shape fixation rate (*R_f_*) and shape recovery rate (*R_r_*) are crucial parameters for evaluating the performance of shape memory. *R_f_* indicates the accuracy with which the temporary shape can be fixed, and *R_r_* quantifies the ability of the polymer to memorize its permanent shape. When performing the measurements in a cyclic SM tension test, *R_f_* and *R_r_* can be calculated using the following equations:

(2) $$R_{f}=\frac{\varepsilon_{u}}{\varepsilon_{m}}\times 100\%$$

(3) $$R_{r}=\frac{\varepsilon_{m}-\varepsilon_{p}}{\varepsilon_{m}}\times 100\%$$

where *ε_m_*, *ε_u_*, and *ε_p_* represent the strain after deformation, at the fixed temporary shape at *T_trans_*, and after recovery, respectively.

The instantaneous recovery velocity *V_r_* can be calculated as the time derivative of the strain, as shown in Equation (4).

(4) $$V_{r}=\frac{\partial \varepsilon }{\partial t}$$

The plot of *V_r_* as a function of temperature reveals the temperature range corresponding to the shape recovery process, which provides a clear thermokinematic view of the shape recovery and helps program for the desired SM effect.

**Figure 7:**
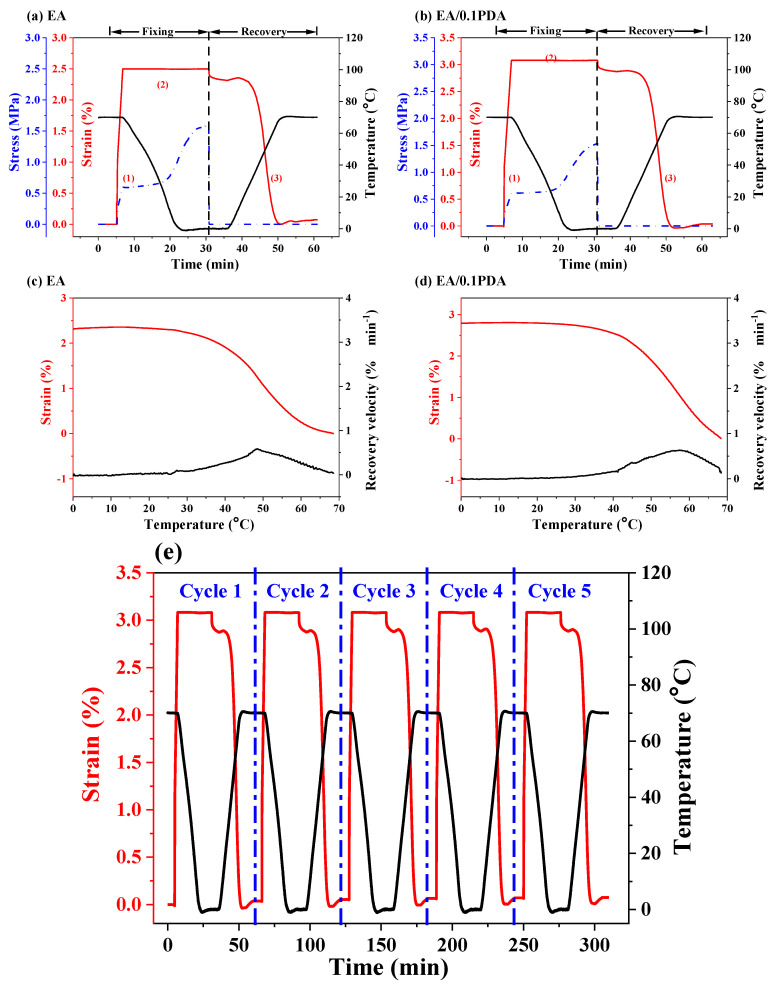
SM thermomechanical cycle tests of UV-cured EA and EA/PDA polymers. (**a**) EA, *ε_m_* = 2.5%, and (**b**) EA/0.1PDA, *ε_m_* = 3.0%. Shape recovery velocity as a function of temperature for (**c**) EA and (**d**) EA/0.1PDA. (**e**) Five consecutive shape memory cycles for EA/0.1PDA. Cooling/heating rate 5 °C/min and N_2_ atmosphere.

Figure 7a,b show good thermal SM performance of the EA sample; in terms of *R_f_* and *R_r_*, which are ~90% and ~100%, respectively. Additionally, EA/0.1PDA EA/PDA with higher PDA concentrations (0.2 and 0.5 wt.%) exhibit similar SM performance (Figure S2). At 70 °C, the storage modulus of EA/PDA polymers is larger than EA, but the *R_f_* and *R_r_* values remain at a similar level, suggesting that PDA did not affect the shape memory effect of EA. Five consecutive deformation, fixation, and recovery cycles were conducted to test the SM performance of EA/0.1PDA over multiple cycles. Figure 7e shows that an identical *R_f_* of ~93% and *R_r_* of ~100% were obtained for each SM cycle under said conditions.

### 3.5. Photothermal Effect and NIR-Triggered SM Behavior

**Figure 8:**
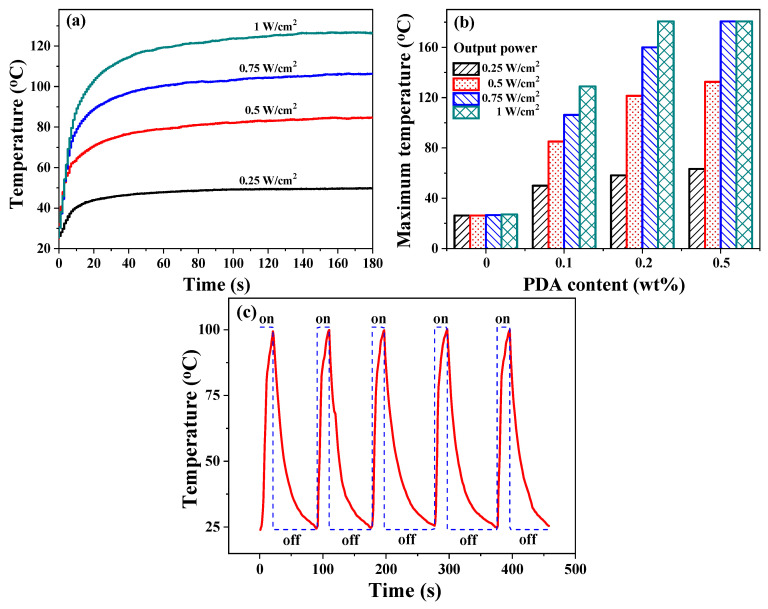
(**a**) Temperature elevation as a function of time for EA/0.1PDA exposed to NIR laser with different light intensity (0.25, 0.5, 0.75, and 1 W/cm^2^). (**b**) The equilibrium temperature rises of EA/PDA polymers as functions of NIR light intensity and PDA concentration. (**c**) Temperature elevation of EA/0.1PDA exposed to 1 W/cm^2^ NIR laser for five cycles.

Figure 8a shows that the heating rate and equilibrium (maximum) temperature of EA/0.1PDA significantly increases as the light intensity increases upon 808 nm NIR irradiation. For instance, the equilibrium temperature only can reach 50 °C in 180 s with a light intensity of 0.25 W/cm^2^. In contrast, when subjected to a light intensity of 1 W/cm^2^, the temperature increases rapidly and reaches 100 °C within a mere 20 s, and the equilibrium temperature can approach ~130 °C in 180 s. In addition, the equilibrium temperature shows a strong dependence on PDA concentration. It can be seen from Figure 8b that the neat EA polymer does not show any temperature variation upon NIR irradiation, whereas EA/0.2PDA exhibits an equilibrium temperature of ~180 °C with a light intensity of 1 W/cm^2^, which is the limit of the infrared thermal imager. The time-dependent temperature of EA/0.1PDA upon NIR irradiation for 20 s (laser on), followed by switching off the NIR laser and naturally cooling to 25 °C, was determined to assess the photothermal stability. Figure 8c shows that the photothermal efficiency does not decline even when the test has been repeated five times, suggesting that the EA/PDA polymer not only possesses high photothermal conversion efficiency, but also exhibits excellent photothermal stability.

**Figure 9:**
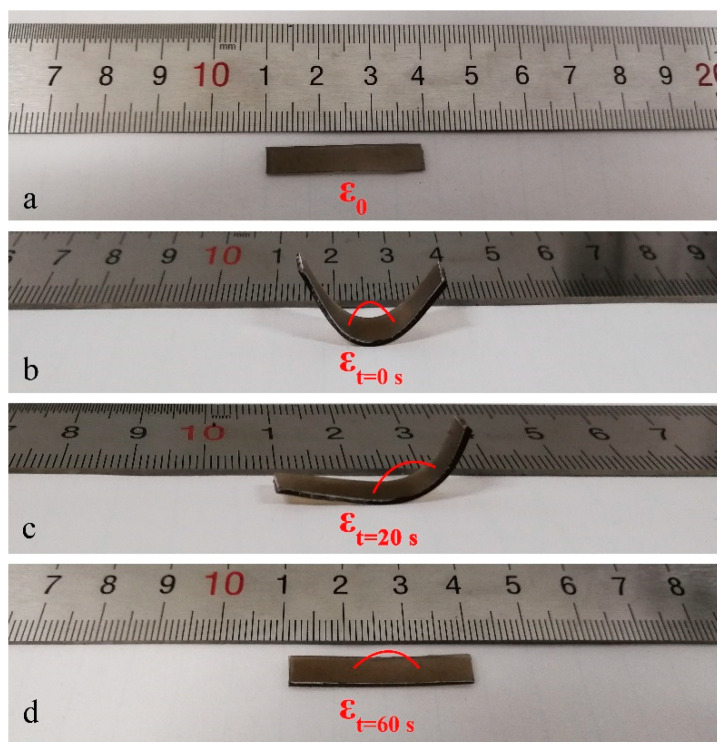
NIR-triggered SM behavior of EA/0.1PDA. (**a**) Original shape. (**b**) Temporary shape before NIR irradiation. (**c**) Temporary shape after 20 s NIR irradiation. (**d**) Shape after 60 s NIR irradiation. NIR output power 1 W/cm^2^.

In order to investigate the SM behavior of EA/PDA polymers triggered by NIR light, an EA/0.1PDA sample with a thickness of 0.78 mm (Figure 9a) was subjected to bending at 70 °C and subsequently cooled to 10 °C to fix the temporary shape (Figure 9b). Figure 9c,d illustrate that when exposed to an 808 nm NIR laser at a power density of 1 W/cm^2^ at 25 °C, the sample undergoes shape recovery, returning to its original unrolled shape. It should be noted that shape recovery occurs in areas exposed to NIR radiation. Thus, the process results in a series of partially recovered temporary shapes, as observed in Figure 9c. Complete shape recovery was achieved once the NIR beam had scanned the entire sample in 60 s, which is much faster than the thermally stimulated liquid crystal elastomers (>10 min) in the literature [18,19]. As a control test, an EA sample was processed to the same temporary shape as EA/0.1PDA. However, when the sample was exposed to the NIR laser under the same condition, no temperature increase or shape recovery was observed.

**Figure 10:**
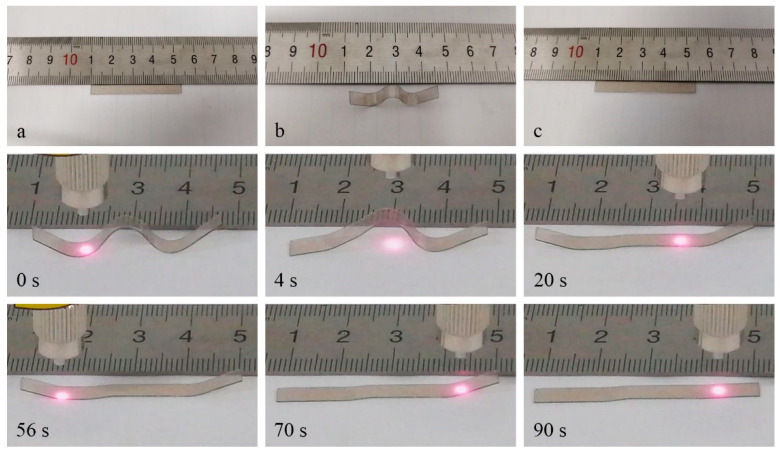
The spatially and temporally controllable NIR-activated SM behavior of EA/0.1PDA. (**a**) Original shape. (**b**) Temporary shape before NIR irradiation. (**c**) Shape after 90 s NIR irradiation. NIR output power 1 W/cm^2^.

As previously discussed, the materials cool down rapidly through heat conduction as the stimulation is ceased, resulting in the cessation of the shape recovery process. This characteristic allows for the control of the shape recovery process and the acquisition of various intermediate shapes. Figure 10 illustrates the outcome of an additional experiment that demonstrates the photo-controlled and spatially selective shape recovery of EA/PDA polymers. A sample of EA/0.1PDA (with a thickness of 0.38 mm) was folded into a compact “W” shape at a temperature of 70 °C and then cooled under strain to a temperature of 10 °C to preserve the temporary shape. The NIR laser was subsequently applied to different areas, resulting in the attainment of multiple intermediate shapes and the completion of the shape recovery process. It is evident that spatially selective shape recovery, facilitated by the rise in temperature of EA induced by PDA’s photothermal effects, can be achieved using NIR. Ordinary heating procedures would not enable this level of control as they would uniformly warm up the whole sample.

### 3.6. Shape Memory Mechanism

**Figure 11:**
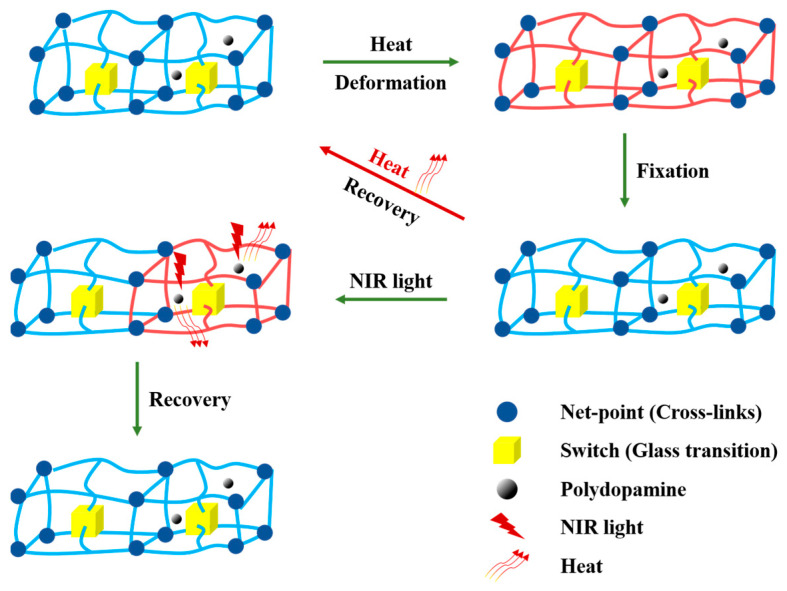
Proposed mechanism model for the thermal responsive and NIR triggered SM behavior of EA/PDA.

According to the above results, it is confirmed that the SM behavior of EA/PDA polymers can be attributed to the photothermal effect, which is caused by the release of heat from PDA when it absorbs NIR (near-infrared) light (Figure 11). As a result of heat release, the temperature of the sample rises above its *T_trans_*. This elevation in temperature is essential for fulfilling the thermal phase transition needed for shape recovery.

## 4. Conclusions

Due to the strong NIR absorption, high photothermal conversion efficiency, and photothermal stability of PDA, a fast and efficient NIR remote-controlled SMP based on PDA and EA has been developed. Upon NIR irradiation, the temperature of the exposed regions can quickly increase to a high *T_trans_*. Shape memory capability can be attained within a brief duration of only 60 s at a low concentration of polydopamine (1 wt.%). The dynamic mechanical properties and thermal resistance of EA polymer were enhanced with the addition of PDA. As an efficient and environmentally friendly photothermal conversion agent, PDA has a great potential for application. EA is a widely used UV-curable polymer. The preparation of EA/PDA polymers has potential application prospects in the field of UV-curing 3D printing technology and smart actuators, such as soft robots or grippers.

## Figures and Tables

**Table 1:** The formulations of EA and EA/PDA polymers.

Sample	Weight Ratio (*w*/*w*)	
	EA	PDA
EA	100	0
EA/0.1PDA	100	0.1
EA/0.2PDA	100	0.2
EA/0.5PDA	100	0.5

**Table 2:** TG and DTG analyses of cured EA and EA/PDA polymers.

Sample	*T_g_* (°C)	*T_di_* (°C)	*T_dmax_* (°C)	*Y_c_* at 580 °C (%)
EA	40	174	413	5.4
EA/0.1PDA	44	200	411	18.7
EA/0.2PDA	46	188	414	13.4
EA/0.5PDA	44	197	413	23.2

## Data Availability

The data that support the findings of this study are available on request from the corresponding authors.

## Supplementary Materials

The following supporting information can be downloaded at: https://www.mdpi.com/article/10.3390/polym15163394/s1, Figure S1: SEM image of the fracture surface of UV-cured EA/PDA polymer; Figure S2: SM thermomechanical cycle tests of UV-cured EA/PDA polymers. (a) EA/0.2PDA, *ε_m_* = 3.0% and (b) EA/0.5PDA, *ε_m_* = 3.0%. Shape recovery velocity as a function of temperature for (c) EA/0.2PDA and (d) EA/0.5PDA.
