# Supplementary material for: Near-Infrared Light Triggered the Shape Memory Behavior of Polydopamine-Nanoparticle-Filled Epoxy Acrylate
Source: Polymers (Basel). 2023 Aug 13;15(16):3394. doi: 10.3390/polym15163394 (PMC10459945; doi:10.3390/polym15163394)
Supplement: Supplementary file 1 [file polymers-15-03394-s001.zip › polymers-2540115-supplementary.pdf]

Supporting Information

Near-Infrared Light Triggered the Shape Memory Behavior of Polydopamine-Nanoparticles-Filled Epoxy Acrylate

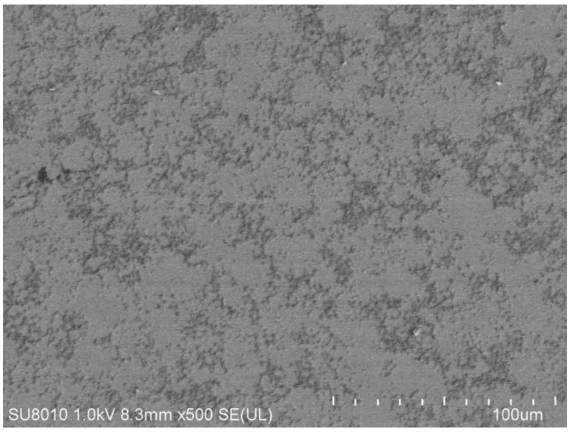

Figure S1. SEM image of the fracture surface of UV-cured EA/PDA polymer.

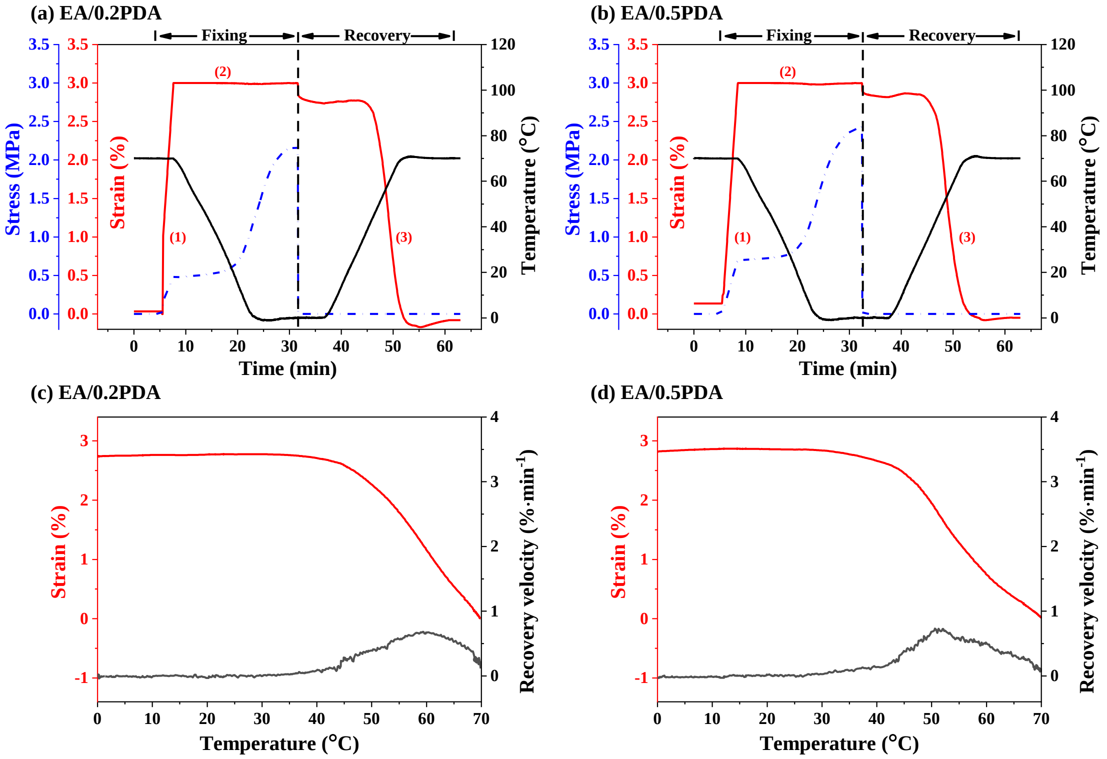

Figure S2. SM thermomechanical cycle tests of UV-cured EA/PDA polymers. (a) EA/0.2PDA, ε_m=3.0% and (b) EA/0.5PDA, ε_m=3.0%. Shape recovery velocity as a function of temperature for (c) EA/0.2PDA and (d) EA/0.5PDA.
